# Probe of Alcohol Structures in the Gas and Liquid States Using C–H Stretching Raman Spectroscopy

**DOI:** 10.3390/s18072061

**Published:** 2018-06-28

**Authors:** Yuanqin Yu, Wei Fan, Yuxi Wang, Xiaoguo Zhou, Jin Sun, Shilin Liu

**Affiliations:** 1 Department of Physics, Anhui University, Hefei 230601, China; yyq@ahu.edu.cn (Y.Y.); sunjin@ahu.edu.cn (J.S.); 2 Hefei National Laboratory for Physical Sciences at the Microscale, iChEM (Collaborative Innovation Center of Chemistry for Energy Materials), Department of Chemical Physics, University of Science and Technology of China, Hefei 230026, China; fw1008@mail.ustc.edu.cn (W.F.); wangyuxi@mail.ustc.edu.cn (Y.W.); xzhou@ustc.edu.cn (X.Z.)

**Keywords:** C–H group, vibrational probe, aliphatic alcohols, conformation, hydrogen bond

## Abstract

Vibrational spectroscopy is a powerful tool for probing molecular structures and dynamics since it offers a unique fingerprint that allows molecular identification. One of important aspects of applying vibrational spectroscopy is to develop the probes that can characterize the related properties of molecules such as the conformation and intermolecular interaction. Many examples of vibrational probes have appeared in the literature, including the azide group (–N_3_), amide group (–CONH_2_), nitrile groups (–CN), hydroxyl group (–OH), –CH group and so on. Among these probes, the –CH group is an excellent one since it is ubiquitous in organic and biological molecules and the C–H stretching vibrational spectrum is extraordinarily sensitive to the local molecular environment. However, one challenge encountered in the application of C–H probes arises from the difficulty in the accurate assignment due to spectral congestion in the C–H stretching region. In this paper, recent advances in the complete assignment of C–H stretching spectra of aliphatic alcohols and the utility of C–H vibration as a probe of the conformation and weak intermolecular interaction are outlined. These results fully demonstrated the potential of the –CH chemical group as a molecular probe.

## 1. Introduction

Vibrational spectroscopy, including infrared and Raman spectroscopy, plays an important role in various research fields due to its sensitivity to molecular structure and the changes in response to external environments. Additionally, vibrational spectroscopy has many attractive characteristics such as being non-destructive and in-situ analysis. This makes vibrational spectroscopy a valuable tool in physics, chemistry, biology, material science, food industry, and security control [1,2,3,4,5,6,7,8]. In vibrational spectra, it is known that the vibrational frequencies are closely correlated with the chemical bonds or chemical functional groups, which are the fundamental units in all molecules. Therefore, one of the important aspects of applying vibrational spectroscopy is to develop the appropriate vibrational probes that can characterize molecular specific information such as the conformation and intermolecular interaction. Many chemical groups have been established as vibrational probes in the literature, including the thiocyanate group (–SCN), azide group (–N_3_), diazo group (–N_2_), the alkyne (–C≡C–), amide group (–CONH_2_), amine group (–NH_2_), carbonyl group (–C=O), hydroxyl group (–OH), –SH group, –CH group, and so on, as summarized in recent several reviews [9,10,11,12,13,14,15,16,17,18,19,20,21,22,23,24,25,26,27]. For example, using the site-specific nitrile group (–C≡N) as a probe of protein structure, it was shown that –CN frequencies could discriminate between the solvent-exposed residues and residues buried in the hydrophobic core of a protein [23]. Recently, Zhang et al. have shown the ability of the diazo group (–N_2_) as a sensor for local hydrogen bonding environments of the diazo model compound, 2-diazo-3-oxo-butyric acid ethyl ester, in different solvents [24]. 

The –CH group has several attractive features when chosen as a probe for specific application. (i) The –CH chemical groups exist widely in organic and biological molecules, which render it possible to become a general tool in molecules; (ii) C–H stretching vibrations not only have relatively high intensity in both infrared and Raman spectra but are also separated from coupling with other vibrational modes; (iii) The C–H probe is sensitive to the local molecular environment. For example, recent theoretical studies have shown that the site-specific C–H stretching vibrations are very sensitive to the backbone conformation of peptide and protein structures [9,10,11,12]; (iv) The C–H probe does not perturb the molecular systems being investigated, which is different from extrinsic molecular probes incorporated into molecules such as the azide group (–N_3_) [20]. As a result of these features, the spectra in the C–H stretching region of 2800–3100 cm^−1^ were widely employed to investigate the molecular structure, intermolecular interactions, gas/liquid interface effect, vibrational energy redistribution mechanism, biological imaging, and identification of polycyclic aromatic hydrocarbons in astrophysics [28,29,30,31,32,33,34,35,36,37,38,39,40,41]. Nowadays, the efforts toward developing C–H vibration as a molecular probe continue to be devoted, as illustrated by several experimental and theoretical research groups [42,43,44,45,46,47]. 

Despite the importance of the C–H stretching vibrational spectra for organic and biological molecules, one challenge of the application of C–H vibrational probe is that the accurate spectral assignments are difficult because of several components in the C–H stretching region such as the symmetric stretching mode (SS), antisymmetric stretching (AS) mode, and Fermi resonances (FR) bands. This easily leads to incorrect assignment even for simple C–H molecules. It is known that the accurate assignment is the first step for understanding the molecular structure and subsequent dynamic process. As an important class of molecules containing –CH groups, aliphatic alcohols, such as methanol (CH_3_OH), ethanol (CH_3_CH_2_OH), 2-propanol (CH_3_CHOHCH_3_) and 1-propanol (CH_3_CH_2_CH_2_OH), are widely used as raw materials, renewable energy and ideal solvents in industry. Academically, they often serve as theoretical and experimental benchmark systems to investigate transient chemical process and the microstructures of complex bimolecular as well as hydrophilic and hydrophobic effects. Although many studies were conducted on the C–H stretching spectra of aliphatic alcohols, the spectral assignments remain unclear from different experimental techniques such as infrared spectroscopy, liquid Raman spectroscopy and sum frequency generation (SFG) spectroscopy [28,48,49,50,51,52,53,54,55,56]. Incomplete assignments have even been used to explain important dynamics such as the intramolecular energy transfer mechanism and the orientation changes of isopropanol in water solution on the gas/liquid interface [28,33]. To better develop the C–H group as a vibrational probe for molecular structures and provide insight into the spectral assignment of more complex organic and biological molecules, we recently launched a project to completely assign the C–H spectra of aliphatic alcohols on the basis of high-resolution gas-phase Raman measurements. Also, we use the C–H vibrational spectra as a probe for the conformation of aliphatic alcohols and weak hydrogen bond in their hydrated state. The advances are outlined here. 

Compared to the broad profiles in infrared spectra, the band shape of gas-phase Raman spectra is narrow and separated, allowing for better resolution in the congested C–H stretching region. Consequently, more new and important spectral features can be revealed. This is due to the fact that the gas-phase molecules are free from molecular interaction that broaden the spectra in the condensed phase or at interface, and more importantly, the Raman selection rules are different from those of the infrared spectra. Unfortunately, Raman photons in the gas phase are generally weak because of the small scattering cross-section, especially in the case that the samples are liquid with low saturated vapor pressure, for example, aliphatic alcohols reported here. Alternatively, one coherent Raman technique, photoacoustic Raman spectroscopy (PARS), offers enhanced sensitivity over the spontaneous Raman scattering in the gas phase. The PARS is a combination of stimulated Raman scattering (SRS) and photoacoustic spectroscopy. It has very high sensitivity and spectral resolution. Additionally, the Raman depolarization ratio is an important parameter to characterize the symmetric category of the vibrational mode. With linearly polarized excitation laser, the ρ value is close to zero for a symmetric mode, whereas it is close to 0.75 for an antisymmetric mode. Therefore, the depolarization ratios can provide further help to assign the observed spectra.

## 2. Materials and Methods

The normal alcohols are purchased from Sigma-Aldrich (Shanghai, China), and the deuterated samples are from the ICON isotope (ICON, Dexter, MI, USA). The water was distilled three times.

### 2.1. Photoacoustic Raman Spectroscopy (PARS)

The principle of PARS can be summarized as follows [57,58]. A stimulated Raman process is generated in the sample when the frequency difference between two laser beams (pump and Stokes beams) matches a Raman-active vibrational transition and they overlap in time and space. As a result, the molecules in the vibrational ground state are transferred to the excited states. Then, the collisions cause the excited-state molecules to relax to the ground state, and the excited energy is inverted to the local heating in the sample molecules, which generates an ultrasonic wave. The signal is detected by a sensitive microphone to obtain the gas-phase Raman spectra. Compared to the conventional Raman measurements in which the Raman scattering photons are directly detected, the sensitivity of PARS is enhanced.

Theoretically, the intensity of the PARS signal is given by

*I* ∝ *σI_P_ I_S_* (cos^2^*θ* + *ρ*sin^2^*θ*), (1)

where *σ* is the Raman scattering section, *ρ* is the Raman depolarization ratio, *I_P_* and *I_S_* are the intensities of the pump and Stokes laser beams, respectively, and *θ* is the cross angle between their polarizations directions. *θ* = 0° means that the polarizations of the pump and Stokes laser beams are parallel to each other, whereas *θ* = 90° means that they are orthogonal to each other. Since the intrinsic property of stimulated Raman scattering is the same as that of spontaneous Raman scattering, the PARS intensity at *θ* = 0° corresponds to the polarized spectrum in conventional Raman, whereas that at *θ* = 90° corresponds to the depolarized spectrum. Similar to a conventional Raman experiment, the intensity ratio of PARS at *θ* = 90° and *θ* = 0° corresponds to the depolarization ratio, as seen from Equation (1). However, for the symmetric vibrational modes, the depolarization ratio is generally small and their depolarized spectrum is weak compared to the polarized one. Then, the determined *ρ* values are highly uncertain with the method of the intensity ratio. In order to overcome this limit, the depolarization ratio is determined by an *I-θ* curve method in present PARS experiments since the PARS intensity is periodically dependent on the cross-angle *θ*. By measuring the *I*-*θ* curve, the depolarization ratio can be accurately determined from a global fitting with Equation (1) [58].

**Figure 1:**
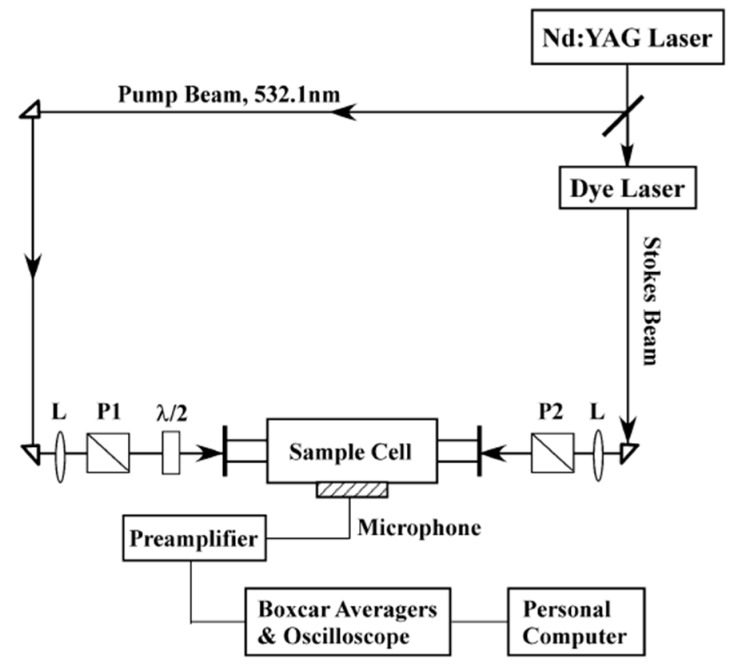
The experimental arrangement for PARS. P1 and P2: Glan prism. L: the lens, Reprinted from Ref. [58].

**Figure 2:**
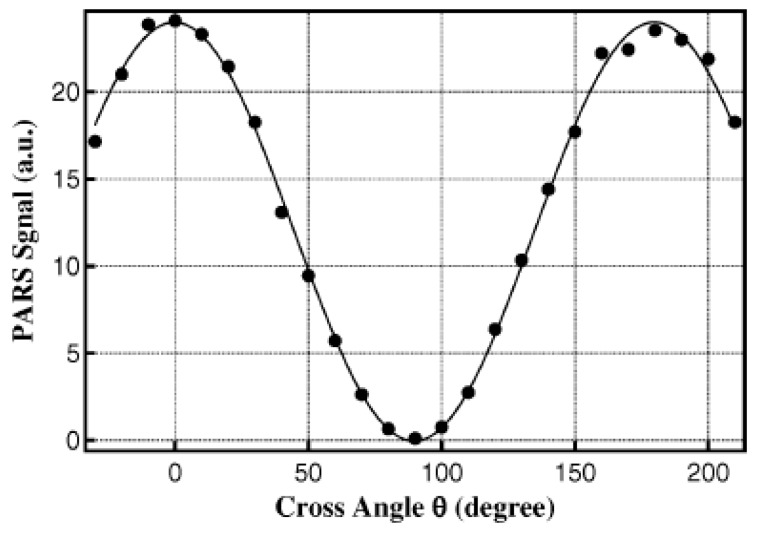
Typical global fitting of the *I*-*θ* curve to determine the depolarization ratio *ρ*. Reprinted from Ref. [58].

The experimental arrangement for PARS is shown in Figure 1 [51]. The light source is from the second-harmonic output of Nd: YAG laser (Pro190, Spectra Physics, Santa Clara, CA, USA), which is split into two parts by a quartz wedge. One is used as the pump beam with ten percent of total energy, and the other is used to pump a dye laser (line width 0.05 cm^−1^, ND6000, Continuum Company, New York, NY, USA) to generate a Stokes beam whose wavelength is tunable. The two laser beams were linearly polarized by two Glan prisms and counter-propagate into the photoacoustic cell with time and space overlapping. The generated Photoacoustic signal is input into an oscilloscope to obtain PARS intensity or averaged by a boxcar integrator to obtain the PARS spectrum. The difference between the wavenumbers of two laser beams only corresponds to the molecular vibrational frequency, i.e., Raman shift. The spectral resolution is about 1.0 cm^−1^ in the present experiment, determined by the convolution of the line width of the pump and Stokes laser beams. In order to measure the depolarization ratio precisely, the polarization of the Stokes beam was fixed in the vertical direction while that of the pump beam was adjusted by a λ/2 half-wave plate to obtain the desired cross angle between the polarizations of two laser beams. A typical photoacoustic Raman signal intensity (*I*) vs. the cross angle (*θ*) is shown in Figure 2 along with the global fitting with Equation (1). The precision of the measured depolarization ratio is checked by the υ_1_ and υ_3_ bands of CH_4_ with an accuracy of 0.005.

### 2.2. Liquid Raman Spectroscopy

For liquid Raman spectra, the samples were excited using a stable cw laser (Coherent, Verdi-V5, 532 nm, power 4 W) and were recorded by a liquid-nitrogen-cooled CCD detector (Spec-10:100B, Princeton Instruments, Trenton, NJ, USA) with a spectral resolution about 2 cm^−1^. The Raman spectrometer is described in detail [59,60,61]. The scattering photons were collected with a configuration of 180° relative to the incident laser beam and were focused with a pair of quartz lenses (f = 2.5 and 10 cm) to be imaged onto the slit entrance of the monochromator. The polarization of incident laser was linear and was controlled with a half-wave plate to obtain the polarized and depolarized Raman spectra. In order to eliminate the polarization dependence of the dispersion grating, one scrambler was used to depolarize the Raman scattering photons. The temperature of the samples was controlled by a heating bath (THD-2006, Ningbo Tianheng Instrument Factory, Ningbo, China). Each spectrum is averaged by ten scans and was corrected for acquisition time.

## 3. Assignment of C–H Stretching Spectra of Aliphatic Alcohols

### 3.1. Methanol

**Figure 3:**
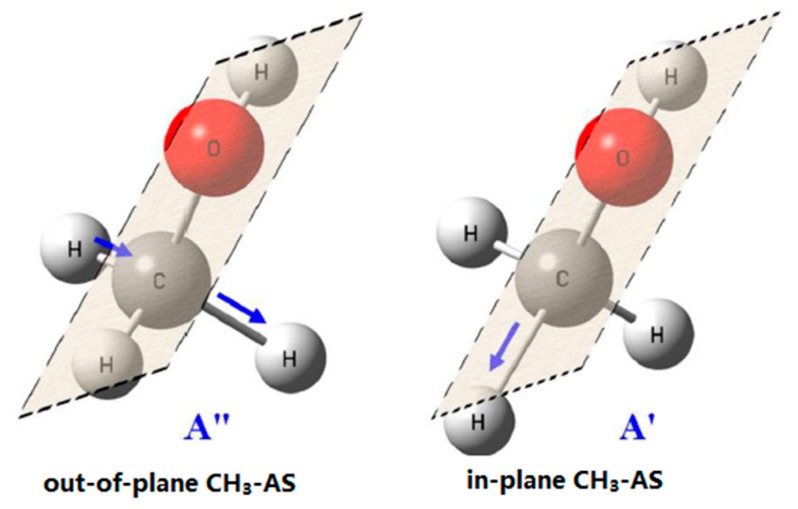
The in-plane and out-of-plane CH_3_ antisymmetric stretching vibrations in methanol. The plane refers to H–C–O–H. Reprinted from Ref. [52].

**Figure 4:**
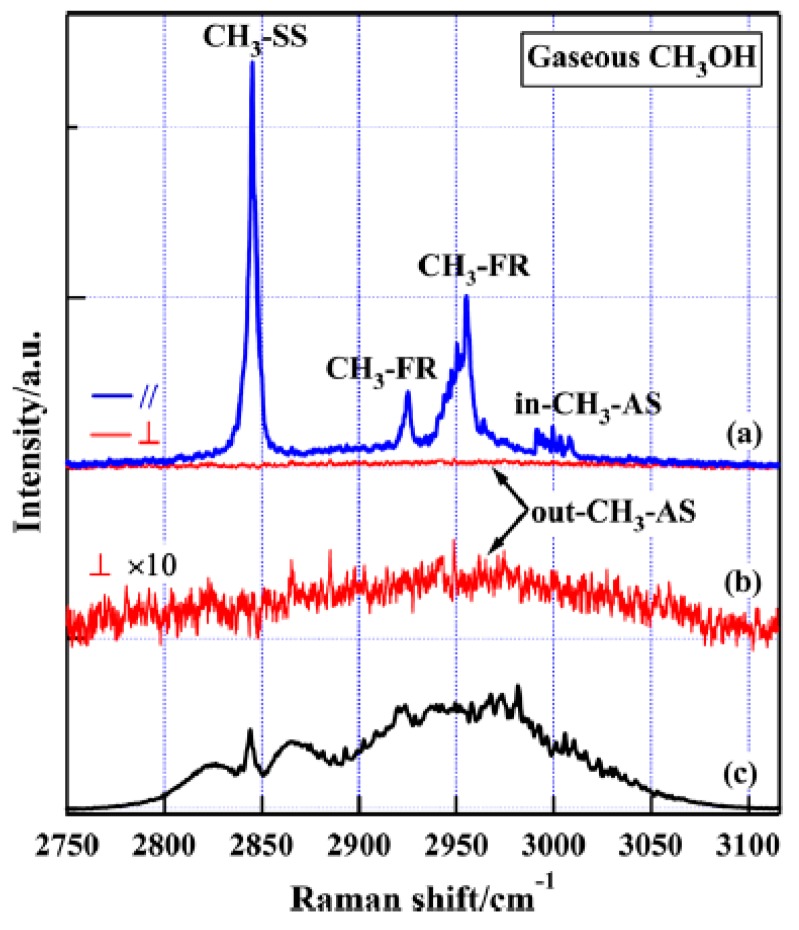
(**a**) Polarized and depolarized C–H Raman spectra of the gaseous methanol in the range 2750–3110 cm^−1^ under parallel (blue line, upper) and perpendicular (red line, lower) polarization configurations of pump and Stokes beams, respectively. CH_3_-SS: CH_3_ symmetric stretching, CH_3_-FR: CH_3_ Fermi resonance, in-CH_3_-AS and out-CH_3_-AS: in-plane and out-of-plane CH_3_ antisymmetric stretching. (**b**) The depolarized spectrum multiplied by ten for the clarity. (**c**) Gas-phase infrared spectrum of methanol in the same region for comparison. Reprinted from Ref. [52].

Methanol is the simplest organic molecule, containing one –CH_3_ group and one –OH group. In general case, –CH_3_ group is treated with C_3V_ symmetry, in which the lengths of three C–H bonds are equal and then CH_3_ antisymmetric stretching vibration is doubly degenerated, such as CH_3_F [62]. In methanol, –CH_3_ group is attached to –OH group, whose symmetry is lowered to C_s_ from C_3v_ symmetry. This leads to the splitting of antisymmetric stretching mode into two bands, in-plane vibration of A′ symmetry (in-CH_3_-AS) and the out-of-plane vibration of A″ symmetry (out-CH_3_-AS), as shown in Figure 3. Therefore, the intrinsic symmetric category of the former is the same as the symmetric mode (A′). The difficulty in clearly distinguishing these two CH_3_-AS bands is one of the main reasons for the inconsistency in previous C–H assignment from different experimental techniques. In addition to two splitting antisymmetric stretching modes, there are symmetric stretching and the overtones of –CH_3_ bending modes in the C–H stretching region. The intensities of overtones are capable of being greatly increased through Fermi resonance with CH_3_ symmetric stretching or in-plane antisymmetric stretching because of the same symmetric category (A′) and close frequencies. For the C–H bands in Figure 4a, the depolarization ratios measured from *I*-*θ* curve method are summarized in Table 1. It can be seen that the value of depolarization ratio is small compared to 0.75, indicating that these modes are from symmetric vibrational modes.

The resolution of gas-phase Raman spectra is significantly improved compared to infrared spectra, as seen from Figure 4. Two new and important spectral features were identified. Firstly, the splitting of in-CH_3_-AS and out-CH_3_-AS vibrational modes was directly distinguished with an interval of 39 cm^−1^ due to different symmetric category, as shown in the polarized and depolarized spectra of Figure 4. Secondly, the out-CH_3_-AS band at ∼2961 cm^−1^ and the CH_3_-FR band at 2955 cm^−1^ overlap each other. Combined with the contrary behavior of the antisymmetric stretching mode in infrared and Raman measurements (weak in Raman but strong in infrared), the inconsistency of spectral assignment in previous studies can be clarified. In infrared spectra, the band at 2960 cm^−1^ was absolutely attributed to the out-CH_3_-AS mode whereas the contribution from CH_3_-FR band was not considered [48,63]. For SFG and Raman measurement, the band at 2960 cm^−1^ was attributed to the out-CH_3_-AS in some literature according to the IR assignment [54,64], but it was assigned to the CH_3_-FR according to polarization measurements [29,65]. However, all above assignments are incomplete. Therefore, the gas-phase Raman spectroscopy demonstrates the capability in resolving complex vibrational spectra. Recently, on the basis of newly identified C–H spectra, Liu et al. investigated the dissociative adsorption behavior and photocatalytic reactions of organic molecules on TiO_2_(110) by analyzing the spectral changes of symmetric stretching, Fermi resonance (FR) and antisymmetric stretching modes on the coverage of methanol [32].

**Figure 5:**
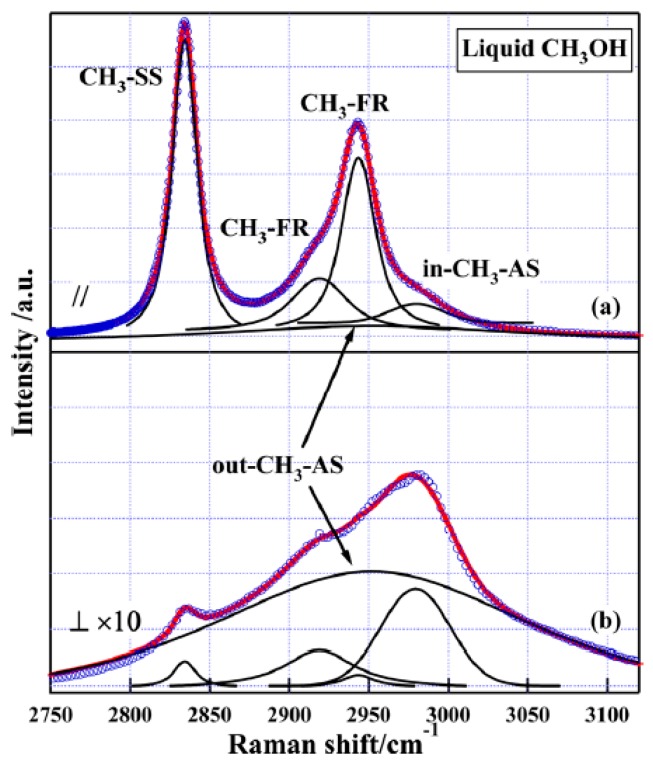
The C–H stretching Raman spectra of liquid methanol. The solid lines are fittings with the Voigt Profiles. (**a**) Polarized spectrum. (**b**) Depolarized spectrum magnified by ten times. Reprinted from Ref. [52].

The C–H stretching Raman bands of liquid methanol are shown in Figure 5 and were assigned according to gas-phase spectral features. It is evident that the spectra are significantly broadened due to the intermolecular interactions in liquid phase. Without detailed knowledge of the gas phase, it is difficult to assign the spectra in the liquid state.

### 3.2. Ethanol

**Figure 6:**
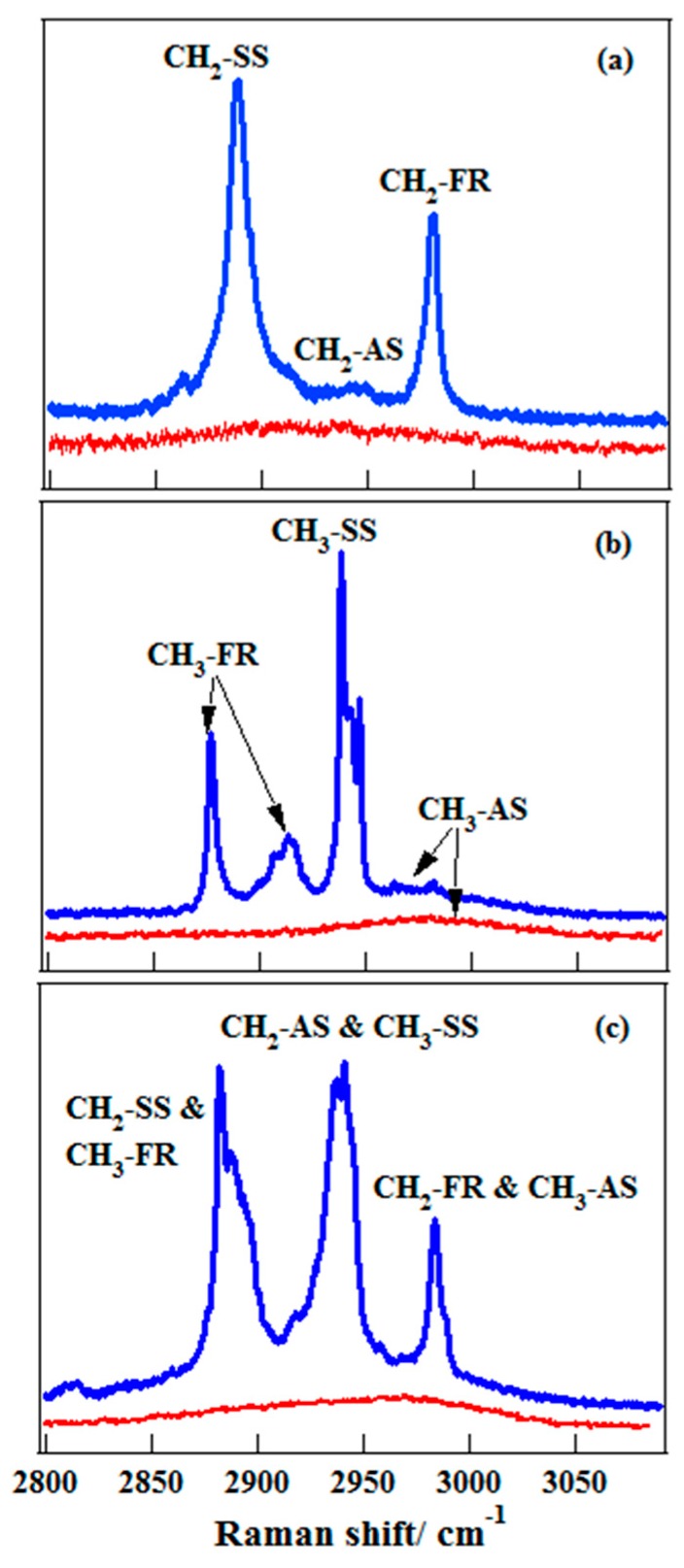
The polarized (top, blue) and depolarized (bottom, red) spectra of ethanol in the C–H stretching region. (**a**) CD_3_CH_2_OH; (**b**) CH_3_CD_2_OH; (**c**) CH_3_CH_2_OH. From Ref. [50].

Ethanol has two different –CH groups, –CH_3_ and –CH_2_. Therefore, its C–H spectra will be more complex compared to those of methanol. Isotope substitution has been demonstrated as a powerful method to unravel the complex spectra. Therefore, the deuterated samples, CH_3_CD_2_OH and CD_3_CH_2_OH, were used to discriminate the spectral contributions of different –CH groups. Figure 6 shows the C–H stretching Raman spectra of gaseous ethanol and their deuterated samples [50]. It can be seen that the spectra of –CH_3_ and –CH_2_ groups show several overlaps, as labeled in Figure 6. Therefore, one must be careful when applying this spectrum to explain the related phenomena. In previous studies, these overlapping spectral features were not revealed [28], and based on those early assignments, the mechanism of vibrational energy transfer within liquid ethanol molecule was investigated [28]. It was shown that the intramolecular vibrational energy transfer occurs firstly from the –OH group to the –CH_2_ group and then from –CH_2_ group to the terminal –CH_3_ group via a through-bond pathway instead of a through-space pathway. However, when considering the spectral overlapping between –CH_3_ and –CH_2_ groups, the energy transfer mechanism should be more complex than expected. From this point, accurate assignment is necessary in the application of C–H spectra as a molecular probe.

Comparing the spectra of CH_3_CD_2_OH (Figure 6b) with those of methanol (Figure 4a), it is interesting to find that the out-of-plane and in-plane CH_3_ antisymmetric stretching vibrations are no longer split in the ethanol molecule but degenerate together in frequency. This is very different from the case for the methanol molecule. Because the –OH group is polar with high electronegativity, the large splitting of CH_3_ antisymmetric stretching vibrations in methanol can be ascribed to the relatively strong interaction between –CH_3_ and –OH groups, which allows the CH_3_ group to deviate from C_3V_ symmetry. However, for ethanol or other long-chain alcohols, the coupling between –CH_3_ and –OH groups decreases rapidly due to the interval of other –CH groups, and then the symmetry of –CH_3_ group is close to C_3V_. In view of the dependence of antisymmetric stretching vibration on the symmetry of –CH_3_ group, its splitting can be used as an indicator of local symmetry of molecules or its geometry changes against external environments. Recently, using the frequency splitting of out-CH_3_-AS and in-CH_3_-AS as a sensor, polarization-dependent SFG experiments revealed that the terminal methyl group favors the C_s_ local symmetry rather than the previously assumed C_3v_ symmetry in the dipalmitoylphosphatidylcholine (DPPC) alkyl tails, and furthermore, the average tilt angles of several functional groups at the interface were determined on the basis of this C_s_ model, including the choline head group, the alkyl tail groups, and the glycerol backbone of DPPC molecules [37]. 

It is necessary to mention that in Figure 6b, the CH_3_-AS band exhibit the weak and broad spectral feature compared to the strong and narrow band shape of symmetric mode. This is because the Raman selection rule is different from that of infrared spectra. Generally, the antisymmetric band is strong in the infrared spectrum whereas it is weak in Raman measurements. On the other hand, the antisymmetric band results from ΔJ = 0, ±1, ±2 rotational transitions whereas the symmetric one is mainly from ΔJ = 0 transitions (Q branch) in the Raman spectrum, leading to the broad spectral feature in antisymmetric band. 

The C–H stretching Raman spectra of 1-propanol and 2-propanol are also reassigned on the basis of the gas-phase Raman measurements [51,53]. In addition to the overlapping spectral features similar to those in methanol and ethanol molecules, we found that the C–H stretching vibration at site-specific carbon atom exhibits sensitivity towards conformational changes of organic molecules. 

## 4. C–H Stretching Vibration as a Conformational Probe

Molecular conformation is a common phenomenon in organic and biological molecules. It plays an important role in many chemical and biological processes. For example, the isomerization of conformation associated with disulfide bonds in proteins can lead to protein denaturation, and the conformation changes of proline is the cause of some diseases [66]. Molecular conformation arises from the free rotation of *σ* single bond in molecules such as C–C, C–O and O–H bonds, leading to different structural arrangements of chemical functional groups within the same molecule. This makes the vibrational frequencies of certain chemical functional groups very sensitive to conformational changes. Historically, the –OH group has been well-established as a conformational sensor for organic molecules, whereas the –CH group is less considered.

### 4.1. 2-Propanol

**Figure 7:**
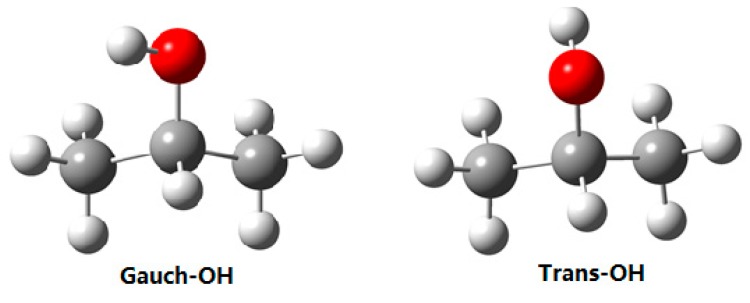
The geometry of two conformers of 2-propanol.

**Figure 8:**
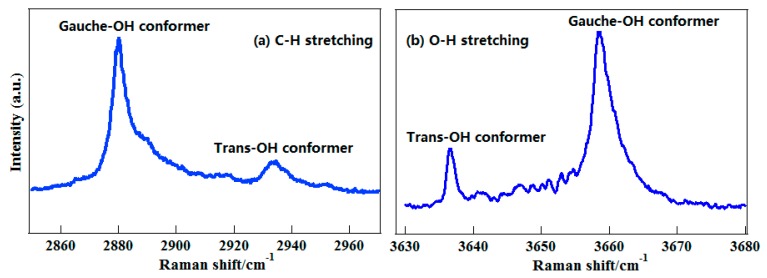
(**a**) The C–H stretching Raman spectra of gaseous CD_3_CHOHCD_3_. (**b**) The O–H stretching Raman spectra of gaseous CD_3_CHOHCD_3_. From Ref. [51].

The 2-propanol molecule has two conformers from different orientations of –OH group, labeled as Gauche-OH and Trans-OH, respectively, as shown in Figure 7. To select the –CH group as a conformational probe, the deuterated 2-propanol, CD_3_CHOHCD_3_, was employed. Figure 8a presents its gas-phase C–H stretching Raman spectra, and as a contrast, the O–H stretching spectra are also plotted in Figure 8b [51]. It is clear that like the O–H vibration, two conformers of 2-propanol can be identified with the C–H vibration.

### 4.2. 1-Propanol

**Figure 9:**
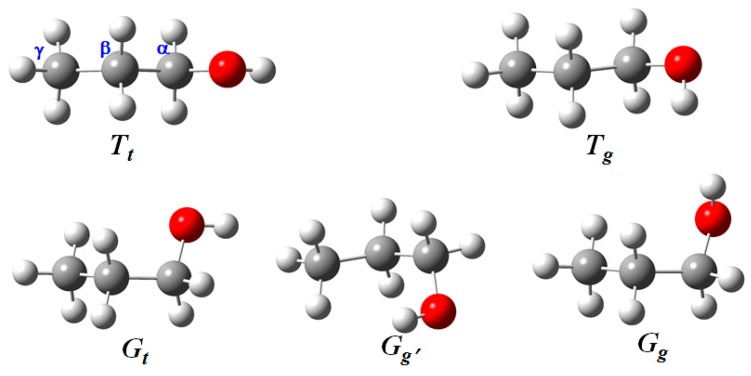
Five conformers of 1-propanol molecule.

**Figure 10:**
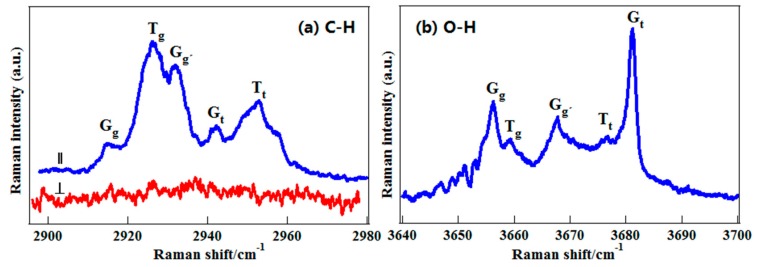
(**a**) The C–H polarized (top, blue) and depolarized (bottom, red) Raman spectra of CD_3_CH_2_CD_2_OH; (**b**) The O–H polarized Raman spectra. From Ref. [67].

The conformation of 1-propanol has been widely investigated using a variety of experimental and theoretical methods, including vibrational spectroscopy, neutron scattering, electron momentum spectrum and rotational spectroscopy as well as quantum chemistry calculation. It has five conformers, G_t_, G_g_, G_g′_, T_g_, and T_t_ resulting from different orientations of two independent dihedral angles, CCCO and CCOH, as shown in Figure 9. The capital letters (G or T) were used to indicate the CCCO dihedral angle, while lowercase letters (t and g) were used to indicate the CCOH. For the notation used here, G and g (Gauche) means that the dihedral angles of CCCO or CCOH equals to 60°, and T and t (Trans) means that the dihedral angles equal to 180°, and the prime (′) indicates that the corresponding dihedral angle has a negative value. Figure 10a exhibits the polarized and depolarized Raman spectra of deuterated 1-propanol, CD_3_CH_2_CD_2_OH, in the region of 2900–2980 cm^−1^ [67]. As seen from Table 1, the measured depolarization ratios of the five intense bands at 2915, 2926, 2932, 2942, and 2952 cm^−1^ are far more less than 0.75, indicating that they are totally from the symmetric stretching vibrations of CH_2_ group at β-carbon position. Therefore, these five bands only correspond to the five conformers of 1-propanol. In previous studies, the results from theoretical predictions have shown that the C–H stretching vibrations can be used to characterize the backbone conformations of protein and peptides [9,10,11,12]. However, the experimental evidence is limited. Here, taking 1-propanol and 2-propanol as examples, the experiments on gas-phase Raman spectra show that the C–H vibrations are very sensitive to molecular conformation and have the potential to act as a conformational probe. This is of great significance since –CH groups are one of the most widely existing function groups in organic and biological molecules.

**Figure 11:**
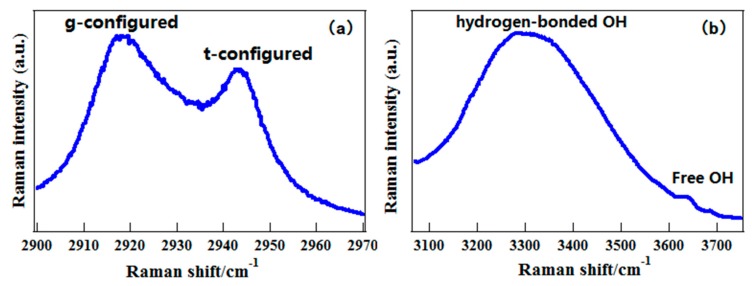
The polarized Raman spectra of liquid CD_3_CH_2_CD_2_OH. (**a**) In the C–H stretching region; (**b**) In the O–H stretching region. Reprinted from Ref. [67].

The O–H stretching Raman spectra of 1-propanol are also plotted in Figure 10b. Like 2-propanol, both –CH and –OH stretching vibrations are sensitive to conformational changes of molecules. To further exhibit the advantage of C–H vibration, both C–H and O–H stretching spectra of liquid CD_3_CH_2_CD_2_OH were shown in Figure 11. Compared to the O–H stretching band, where the conformational features are erased with a single band, the information on conformations of liquid 1-propanolcan is still partly retained in the C–H stretching band. This is to say that the C–H vibration has better adaptability in different environments. According to the gas-phase spectral features, the band at 2943 cm^−1^ was assigned to two conformers of liquid 1-propanol with trans-OH group, T_t_ and G_t_, and denoted as t-configured conformers. The strong band at 2920 cm^−1^ was assigned to three conformers of liquid 1-propanol with the gauche-OH group consisting of T_g_, G_g_ and G_g__′_, and denoted as g-configured conformers. With these two characteristic spectral lines as a probe, the conformational preferences of 1-propanol in pure liquid state and aqueous solution were investigated, whereas those cannot be investigated with the O–H vibration. It was shown that the water molecule can further stabilize the trans-OH conformation when forming hydrogen-bond structures with 1-propanol in their solutions [67]. These results fully demonstrate the advantage of the C–H chemical group as a conformational probe in different environments.

## 5. Characterization of Weak Hydrogen Bonds in Methanol-Water Solutions

Molecular vibrational frequencies depend on environmental factors such as the interactions between the solute and solvent molecules. The hydrogen bond is one of the most important noncovalent interactions in molecules, playing a key role in molecular structures, physical and chemical properties, biological process, and material science. The standard hydrogen bond is formed between X–H and Y through electrostatic interaction, i.e., X–H···Y, where H is the hydrogen atom, X and Y are highly electronegative atoms such as F, N, and O. The standard hydrogen bond leads to the red shifting of X–H stretching frequency accompanying an enhancement of its vibrational intensity. In recent years, another unusual kind of X–H···Y hydrogen bond has attracted extensive interest, where X is an atom with weak electronegativity such as C and Si. Another unusual kind of X–H···Y hydrogen bond follows the contrary trend: blue shifting of X–H stretching frequency and a decrease in spectral intensity [68,69,70]. The evidence is accumulating to show that the blue-shifted hydrogen bond plays a role in supramolecular design, crystal packing, and structure–activity relationship in both biological and chemical processes. However, the blue-shifted hydrogen bond is weak compared to the red-shifted one. 

**Figure 12:**
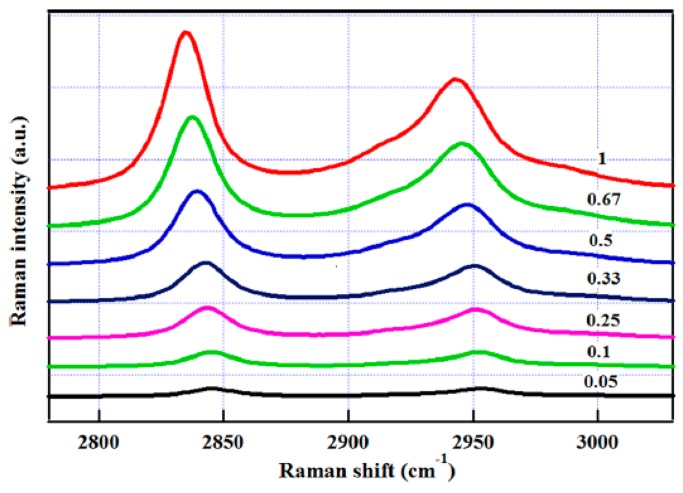
The C–H stretching Raman spectra of methanol–water mixtures with the decrease of the mole fraction of methanol from top to bottom. Reprinted from Ref. [71].

**Figure 13:**
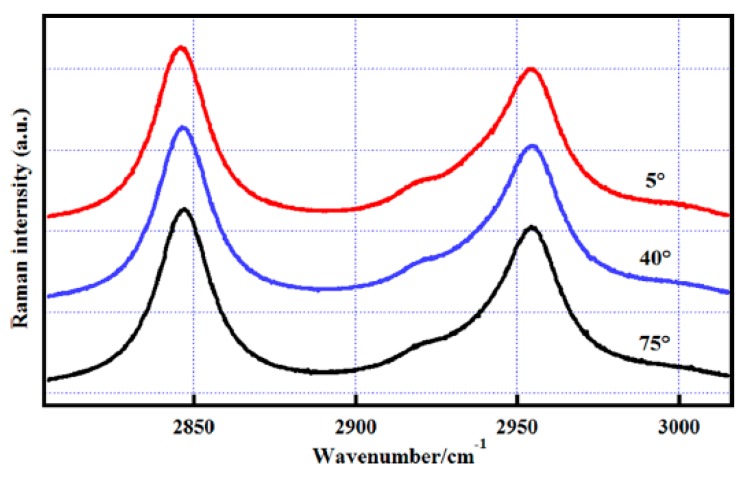
The C–H stretching Raman spectra of the methanol–water mixture at a mole fraction of methanol equal to 0.1 at 5, 40 and 75 °C. Reprinted from Ref. [71].

Among the blue-shifted hydrogen bonds, the C–H···O is the most common one. In methanol-water solution, the frequency of CH_3_ stretching vibration was shifted to higher wavenumbers due to the increase in water concentration, as shown in Figure 12 [71]. This is consistent with the expected behavior for the formation of a blue-shifted hydrogen bond between the methyl group of methanol and the O atom of water (C–H···O). However, the theoretical calculation from a cluster model indicated that this C–H blue shift can also be indirectly caused by a normal hydrogen bond O–H···O in methanol-water solution [71]. To distinguish the contribution of O–H···O from that of C–H···O and provide evidence for presence of C–H···O in methanol-water solution, the temperature-dependent C–H spectra are recorded in the range of 5–75 °C at the concentration of 1% solution (Figure 13). Expectedly, the C–H spectrum blue shifts further to higher wavenumber with 1–2 cm^−1^ with the increase in temperature, indicating that the C–H···O interaction contributes to the C–H blue shift in the methanol-water solution and the normal hydrogen bond O–H···O cannot be fully responsible for it. The reasons are as follows. The temperature plays a role in destroying the red-shifted hydrogen bond, leading to the blue shift of O–H stretching vibration at high temperatures. Therefore, if the C–H blue shift is totally from the normal hydrogen bond O–H···O, the C–H stretching frequency should be red-shifted when the temperature increases, which is contrary to the O–H vibration. However, this is not the case observed in temperature-dependent spectra, as seen from Figure 13. Therefore, using C–H spectra, the experimental evidence for the presence of an abnormal hydrogen bond C–H···O in methanol-water solution was identified. This can be expected to increase the understanding of the interaction process of organic and biological molecules in the aqueous environment. This is especially important when considering that most biological molecules play a function in the water solution. 

## 6. Conclusions

The vibrational frequency of chemical functional groups is one of the most accurately measurable spectroscopic properties of molecules in various environments such as gas, liquid and solid states, and it is sensitive to the changes of external environments. Accordingly, infrared absorption and Raman scattering spectroscopy has become a powerful tool to investigate molecular structure, conformation and dynamical processes of interest. To fully use chemical groups for qualitative and quantitative analysis, a lot of vibrational probes have been developed, such as the amide group –CONH_2_, hydroxyl group –OH, Sulphur group –SH, amino group –NH_2_, and –CH group and so on. In addition to these native vibrational probes, in recent years, there is an increased interest in the utility of various extrinsic probes such as azide (–N_3_) and nitrile (C≡N) groups that can be incorporated into biomolecules in a site-specific way to investigate, for example, the local electrostatics and dynamics of proteins and DNA [19]. Compared to extrinsic molecular probes, the C–H probe does not perturb the molecular systems being investigated and is sensitive to the molecular local environment. In this paper, we briefly outline our recent advancements in the development of a C–H chemical group as a probe of alcohols, including the complete spectral assignment in the C–H stretching region, identification of molecular conformation, and characterization of weak intermolecular interactions in the hydrated process. In addition to the C–H probe, the isotope-substituted carbon-deuterium (C–D) probe has recently been applied, increasing the investigation of various process since it falls into the so-called “transparent window” spectral region (2000–2400 cm^−1^). For example, the kinetics of proton transfer in the model compound malonaldehyde was monitored with a C–D reporter by simulating two-dimensional infrared (2D IR) spectroscopy computationally [15]. Additionally, using C–D bonds as probes, the population and dephasing dynamics of selectively excited C–D bonds in a deuterated amino acid were characterized and the intramolecular vibrational energy redistribution (IVR) within proteins was revealed [13]. These results fully demonstrated the capability of C–D or C–H vibration as a molecular probe in various systems. 

## Figures and Tables

**Table 1:** The measured depolarization ratios for symmetric CH bands.

Molecules	Band Position/cm^−1^	*ρ*	Assignment
CH_3_OH	2845	0.01	CH_3_-SS
	2925	0.09	CH_3_-FR
	2955	0.038	CH_3_-FR
	3000	0.150	in-CH_3_-AS
CH_3_CD_2_OH	2878	0.007	CH_3_-FR
	2913	0.051	CH_3_-FR
	2938	0.015	CH_3_-SS
CD_3_CH_2_OH	2888	0.038	CH_2_-SS
	2981	0.045	CH_2_-FR
CH_3_CH_2_OH	2882	0.051	CH_3_-FR & CH_2_-SS
	2938	0.083	CH_3_-SS & CH_2_-AS
	2983	0.151	CH_2_-FR & CH_3_-AS
CD_3_CH_2_CD_2_OH	2915	0.230	Tt-*β*-CH_2_-SS
	2926	0.108	Gt-*β*-CH_2_-SS
	2932	0.113	G_g__′_-*β*-CH_2_-SS
	2942	0.210	T_g_-*β*-CH_2_-SS
	2952	0.096	G_g_-*β*-CH_2_-SS
